# LncRNAs *NEAT1*, *HOTAIR*, and *GAS5 expression in hypertensive* and non-hypertensive associated cerebrovascular stroke patients, and its link to clinical characteristics and severity score of the disease

**DOI:** 10.1016/j.ncrna.2022.10.004

**Published:** 2022-11-02

**Authors:** Marwa A. Ali, Olfat G. Shaker, Abeer A. Khalifa, Eman M. Ezzat, Hany Ahmed Elghobary, Tamer Sayed Abdel Mawla, Ahmed Fathy Elkhateeb, Ahmed Magdy Abdelrahman Elebiary, Azza Mohamed Elamir

**Affiliations:** a Department of Medical Biochemistry and Molecular Biology, Faculty of Medicine, Fayoum University, Fayoum, Egypt; b Department of Medical Biochemistry and Molecular Biology, Faculty of Medicine, Cairo University, Cairo, Egypt; c Department of Physiology, Faculty of Medicine, Zagazig University, Egypt; d Department of Internal Medicine, Faculty of Medicine, Fayoum University, Egypt; e Department of Clinical and Chemical Pathology, Faculty of Medicine, Cairo University, Cairo, Egypt; f Department of Critical Care, Faculty of Medicine, Fayoum University, Fayoum, Egypt; g Department of Physiology, Faculty of Medicine, Fayoum University, Fayoum, Egypt

**Keywords:** LncRNA, NEAT1, HOTAIR, GAS5, Cerebrovascular stroke

## Abstract

**Background:**

Cerebrovascular stroke (CVS) is a potentially fatal disease. The most common risk factor for CVS is hypertension.

**Aim:**

While most studies in the field have focused on the functional roles of long noncoding RNAs (lncRNAs) NEAT1, GAS5, and HOTAIR in CVS, less attention has been paid to their clinical relevance to stroke incidence and prognosis. Also, a link has not yet been made between these lncRNAs and hypertension, our study aim was to investigate whether the expression of these lncRNAs differed between CVS with and without hypertension, as well as to compare each group to controls.

**Method:**

In total, 181 CVS patients were enrolled, including 91 chronic hypertensive patients with stroke, 90 stroke patients without hypertension, and 51 control subjects. blood samples were collected on the day of recruitment from patients with CVS and controls. Real-time qRT-PCR was used to detect the expression of target lncRNAs in serum.

**Results:**

When compared to controls, there was a statistically higher level of lncNEAT1 in each case group (median (IQR) = 3.68 (1.35–7.35) and 3.05 (0.95–6.45) for the hypertensive and non-hypertensive groups, respectively, with a significantly higher level in the hypertensive group (P = 0.04). When compared to controls, lncHOTAIR was significantly downregulated in all case groups (medians in hypertensive and non-hypertensive patients were 0.13, and 0.34, respectively), with a significantly lower level in the hypertensive group (P = 0.05). LncGAS5 levels in patients were significantly lower (median (IQR) = 0.16 (0.02–0.55) and 0.25 (0.03–0.99) for the hypertensive and non-hypertensive groups, respectively) compared to controls, with a significantly lower level in the hypertensive group (P = 0.02). There was a significant positive correlation between NEAT1 and GAS5, but a significant negative correlation between each with HOTAIR in both patients' groups. We also detected a significant negative correlation between each NEAT1 or GAS5 and NIHSS score while a significant positive correlation between HOTAIR and NIHSS. ROC curve analysis for *GAS5* was able to differentiate patients with CVS hypertensive from patients with CVS non-hypertensive.

**Conclusion:**

Patients in each case group had statistically higher levels of NEAT1 and lower levels of HOTAIR and GAS5 compared to control levels, with higher significant NEAT1 but lower significant HOTAIR and GAS5 in the hypertensive group. Therefore, lncRNAs NEAT1, HOTAIR, and GAS5 could be used as diagnostic and prognostic biomarkers of CVS that correlate with NIHSS score and could produce a novel target for CVS therapy.

## 1 Introduction

Cerebrovascular stroke (CVS) is a sudden decrease in cerebral blood flow that results in brain injury or infarction. Ischemic stroke is one of the major causes of physical disability and mortality worldwide [1]. Diseases of the circulatory system, including stroke, are the primary causes of death in developing countries. Hypertension is the most common risk factor for stroke, as it is observed in approximately 64% of patients with stroke [2]. The persistent high intravascular pressure caused by hypertension is correlated with stroke pathogenesis [3].

Long non-coding RNAs (lncRNAs) are defined as transcripts of more than 200 nucleotides that have been identified as important regulators of several biochemical functions [4]. Recent research discovered that ischemic stroke patients and healthy donors had different lncRNA expression patterns that have been discovered as being implicated in the molecular pathways underlying the ischemic cascade [5].

The lncRNA nuclear enriched abundant transcript 1 (NEAT1) was recognized as a modulator of the stability of paraspeckle nuclear bodies. NEAT1 has been extensively studied as a cancer regulator [6], and a number of studies have recently examined the link between NEAT1 and vascular and ischemic diseases. For example, two separate 2021 studies reported that the silencing of NEAT1 alleviates preeclampsia and pulmonary hypertension via regulating the miR-485-5p/AIM2 axis or miR-34a-5p/KLF4 axis respectively, [7,8].

The lncRNA growth arrest-specific 5 (GAS5) has been proposed as a tumor-inhibiting factor in different types of cancers [9]. Recent studies have also correlated GAS5 with vascular disease. For example, GAS5 induces apoptosis in vascular smooth muscle cells during atherosclerosis, which results in vascular narrowing [10]. Similarly, GAS5 plays an essential role in vascular remodeling during arterial hypertension, and it is a crucial regulator of apoptosis and proliferation of VSMCs by inhibiting β-catenin signaling and/or miR-21 [11,12]. GAS5 is also involved in ischemic stroke progression by functioning as a competing endogenous RNA for miR-137, which regulates the Notch1 signaling pathway [13].

The lncRNA HOX transcript antisense intergenic RNA (HOTAIR) has been shown to play a role in the development and progression of several complex disorders [14]. HOTAIR was discovered to control the development of preeclampsia by inhibiting miR-106 in hypertensive diseases and vascular remodeling [15]. Furthermore, HOTAIR amplification was shown to reduce viability and increase apoptosis in ox-LDL-treated VSMCs by its effect on the miRNA-130b-3p/PPAR axis [16]. In the case of cerebral and cardiac occlusive diseases, HOTAIR, according to Yang et al., 2016, may enhance ischemic stroke caused by hypoxia by upregulating the NADH oxide 2 (NOX2) enzyme [17].

While most studies in the field have focused on the functional roles of these lncRNAs in CVS, less attention has been paid to their clinical relevance to stroke incidence and prognosis. Also, a link has not yet been made between these lncRNAs and hypertension, which is the leading risk factor for stroke. As a result, the aim of this study was to clarify whether the expression of these LncRNAs differs in patients with hypertensive vs. non-hypertensive CVS, and if there are differences between each group when compared with a control group, in order to explore whether they could be used as a therapeutic target in CVS patients, particularly those with hypertension and to assess their utility as diagnostic and prognostic biomarkers for stroke, as well as their relationship with clinical, laboratory characteristics, and severity scores.

## 2 Methods

### 2.1 Participants and ethical approval

**Fig. 1:**
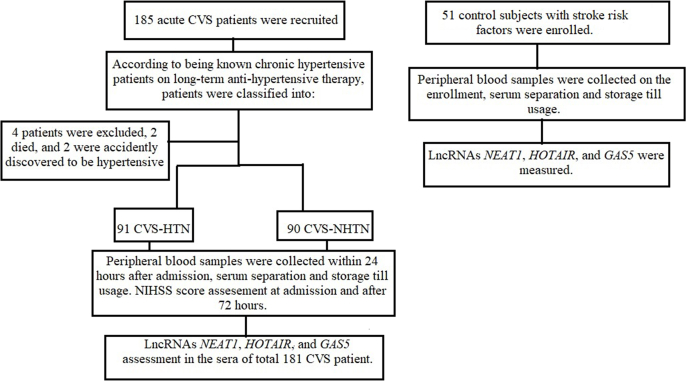
Follow of patients and controls.

The current case-control study included a total of 181 ischemic stroke patients (Fig. 1). They were selected in sequential order from Fayoum University Hospital's Intensive Care Units in the Internal Medicine and Neurology Departments between Jan 2022 and June 2022. According to American Stroke Association guidelines [18], patients were diagnosed with acute cerebrovascular stroke (CVS). The inclusion and exclusion criteria used to select the subjects are shown in Table 1.

**Table 1:** Inclusion and Exclusion criteria used to select the patients.

Inclusion criteria	Exclusion criteria
- Recent onset of ischemic stroke according to patient medical records, and no cerebral bleeding.	- Patients who were accidently discovered to be hypertensive during a clinical examination,
- Over the age of 18.	- Patients who had a concurrent active inflammatory disease.
- With a computed tomography (CT) scan or magnetic resonance imaging (MRI) that confirmed the diagnosis.	- With a history of cancer or malignancy anywhere in the body, or who have received immunosuppressive therapy in the year preceding the study.
- Accepted to participate in the study.	- Patients suffering from hemorrhagic stroke or other neurological disorders; brain trauma or congenital cerebral aneurysms.
	- Patients who died within 24 h of enrollment.
	- Pregnant and lactating females

Our patients are classified as chronic hypertensive patients with stroke and stroke patients without hypertension based on the history of known chronic hypertensive patients on long-term anti-hypertensive medications (according to European guidelines, hypertension is defined as a BP ≥ 140/90 mmHg) [19]. Fifty-one subjects, with age and gender-matched to case groups, were recruited from Internal Medicine outpatient clinics in the same hospital with a history of at least two stroke risk factors, including current smoking, a disturbed lipid profile, high cholesterol, diabetes mellitus, hypertension, and a lack of physical activity. Controls were excluded if they had a history of stroke, malignancies, ischemic heart diseases, rheumatic heart diseases, atrial fibrillation, valve replacement, atherosclerosis, carotid stenosis, current active infection, or were a pregnant or lactating woman. Informed consent was obtained from all participants after approval of the study protocol by the El Fayoum Ethical Committee and the study took registration no (R208- session 89). This study is carried out according to the Declaration of Helsinki [20].

### 2.2 Classifications of participants, data collection, and clinical examination

Following recruitment, a complete history of risk factors and concurrent illness was recorded including age, gender, hypertension, history of diabetes mellitus (DM), smoking, rheumatic heart disease (RHD), atrial fibrillation (AF), ischemic heart disease (IHD), valve replacement procedure (VR), current therapy, history of previous stroke, or any other illness. All participants' blood pressure was measured three times and the mean was calculated. All participants also underwent laboratory tests such as hemoglobin concentration (HB), thyroid profile tests (freeT3, freeT4, and TSH), lipid profile (Total cholesterol (TC), triglycerides (TG), LDL, HDL), kidney function tests (urea, creatinine), fasting blood glucose (FBG), and 2 h postprandial blood glucose (2hsPPBG). All patients underwent CT and echocardiography. ECG and carotid doppler were used to rule out heart disease, atherosclerosis, and carotid artery stenosis, respectively.

### 2.3 Severity scores

Severity scores include The National Institutes of Health Stroke Scale (NIHSS), a measure used by healthcare practitioners to objectively evaluate the disability caused by a stroke, which consists of 11 items with the highest possible score being 42, while the lowest conceivable score is 0 [21]. NIHSS was done after 24 h and after 72 h of stroke onset. The Modified Rankin Scale (mRS) assesses impairment in stroke patients and is used to compare recovery and the degree of ongoing disability over time. A score of 0 indicates no impairment, a score of 5 indicates disability that needs continual care for all requirements, and a score of 6 indicates death [21]. On discharge, mRS was performed.

### 2.4 Target lncRNAs selection

Because the three target lncRNAs (NEAT1, GAS5, and HOTAIR) are present in detectable amounts in circulation [6,13,14], their circulatory levels are differentially expressed in a specific manner in relation to diseases with a focus on hypertensive diseases (arterial hypertension, pulmonary hypertension, and preeclampsia) [7,8,11,15], with CVD (MI, AF) [7,8,14,15], and with CVS [6,11,17], they participate in intracellular communication and seem to affect vasculature in studies combined both human and mice models [8,14]. They are associated with hypertension pathways, hypertensive induced stroke pathways, and vascular occlusive disorders molecular pathways [2], but no single study investigates their expression in patients who suffered from CVS and are well known to be chronically hypertensive patients. Thus, we selected the three target lncRNAs (*NEAT1, GAS5, and HOTAIR*) and hypothesized that they might play an important role in the therapeutic plan of CVS especially if they are related to hypertension (a major risk factor for stroke).

### 2.5 Sample collections, RNA extraction, and cDNA synthesis

Peripheral blood (PB) samples were collected on the day of recruitment from patients with CVS and controls. A venous blood sample of 5 mL was delivered to a plain tube. After 15 min of coagulation, the serum was separated by centrifugation at 4000×*g* for 10 min. Serum samples were immediately stored at −80 °C until use. We extracted total RNA from the sera using the MiRNeasy Serum/Plasma extraction kit (Qiagen, Hilden, Germany) after adding the QIAzol lysis reagent according to the manufacturer's instructions; in a new collection tube, we pipetted 200 μl serum to 1000 μl QIAzol Reagent (about 5 vol of samples amount). After mixing well by vortexing, we added an equivalent volume of chloroform (200 μl) to the initial sample in the tube and vortexed for 15 s. Following centrifugation, the specimen separated into three parts: an uppermost aqueous solution containing RNA, whitish interphase, and a bottom, pink organic layer. We transferred the topmost aqueous solution (almost 600 μl) to a clean collecting tube, added 1.5 vol of 100% ethanol (900 μl), and carefully mixed it by pipetting up and down numerous times. We used the RNeasy spin column (supplied by the kit) to purify RNA from other debris in two phases; for each we used 700 μl of the sample, centrifuged at 8000×*g* for 15 s, and discarded the flow-through. We used the washing buffers (RWT, RPE, and 80% ethanol) to wash the RNA as follows: first, we added 700 μl RWT to the spin column, closed the lid, centrifuged at 8000×*g* for 15 s, discarded the flow-through, then repeated this step with 500 μl RPE followed by 500 μl 80% ethanol, after discarding the flow-through, we put the spin column to new 2 ml tube, opened the lid, and centrifuged at full speed for 2 min for dehydration, we used RNase-free water for elution.

The NanoDrop® (ND)-1000 spectrophotometer (NanoDrop Technologies Inc., Wilmington, DE, USA) was used to determine the extracted RNA purity and concentration.

For the long noncoding RNA analysis, total RNA was reverse-transcribed in a total volume of 20 μL/reaction using the RT2 first strand kit (Qiagen, Maryland, MY, USA) following the manufacturer's recommendations.

### 2.6 Real-time quantitative qPCR for measurement of target lncRNAs expressions in sera of participants

Previously, target lncRNAs in serum were measured [[22], [23], [24]]. The levels of the lncRNAs NEAT1, HOTAIR, and GAS5 were determined using quantitative real-time PCR (RT-qPCR). RT-qPCR was performed using the Rotor-gene Q real-time PCR system (Qiagen, USA). We used the RT2 SYBR Green PCR kit (Qiagen, Germantown, MD, USA), a predesigned specific primer for each lncRNA, and the housekeeping gene (GAPDH) [36] were obtained from (Qiagen, Valencia, CA, USA), NEAT1 (Catalog no: 330701 LPH15809A, Accession no: NR_028272.1), GAS5 (Catalog no; 330701LPH11340A, Accession no, NR_002578.2), HOTAIR (Catalog no; 330701LPH07360A Accession no; NR_003716.3), and GAPDH housekeeping gene (Catalog no: 330701 LPH31725A, Accession no: ENST00000496049.0) to execute the PCR reactions. The PCR cycling procedure for quantifying lncRNAs begins with a 10-min incubation at 95 °C, followed by 40 cycles at 95 °C for 15 s and 60 °C for 60 s. The 2^−ΔΔCt^ equation was used to calculate the serum fold changes of NEAT1, GAS5, and HOTAIR. Non-coding RNAs with a fold change (FC) less than one were downregulated, whereas those with an FC more than one were upregulated [25]. The controls FC values were set as one.

### 2.7 Sample size calculation

We used a sample size of 90 cases, and we examined the power of the sample by G∗power software for the different tests of two tails used in the statistical analysis (F test, Z tests as regression, Spearman correlation test) using the medium effect of Cohen, the power of sample ranged from 0.889 to 0.999), the critical F was 3.85.

### 2.8 Statistical analysis

Data was presented in numbers and percentages, median and interquartile ranges; (IQR), and mean ±SD (standard Deviation), The SPSS version 22 (SPSS Inc) was used to analyze data. Median and range were calculated for the quantitative data. when variables were not normally distributed, the Mann–Whitney-U test (2 groups) or Kruskal Wallis test (more than 2 groups) was used in comparing groups. Otherwise, the one-way ANOVA (for comparing the three groups) or the independent-T test (comparing the hypertensive and non-hypertensive CVS patients’ groups) was used. Chi-square (χ2) was performed to detect the significance of the qualitative data, if the expected frequency is < 5, the exact test was used instead. Spearman correlation was done to explore the association between *NEAT1, HOTAIR*, and *GAS5* and the clinical parameters. Multivariable linear regression analysis with NIHSS score as a dependent factor was done. The receiver operating characteristic (ROC) curve analysis was done to detect the sensitivity and specificity of *NEAT1, HOTAIR, and GAS5* regarding the discrimination between CVS cases with or without hypertension and differentiation between CVS patients with healthy control subjects. All the results were interpreted its significance by considering p ≤ 0.05 is significant.

## 3 Results

### 3.1 Basic characteristics and laboratory parameters of three studied groups

This case-control study included 181 patients who presented with acute cerebrovascular stroke (CVS), 91 of them known to be hypertensive (CVS + HTN) and 90 were non-hypertensive patients (CVS + NHTN). Fifty-one participants volunteers were involved as a control group. Patients with CVS and HTN had a mean age of 59.4 ± 8.55 years, CVS patients and NHTN had mean age of 56.71 ± 11.53 years and 51 controls had a mean age of 55.66 ± 10.08 years (Table 2). There were 72 (79.13%) males in the hypertensive group, 72 (80.0%) males in the non-hypertensive group, and 40 (78.43%) males in the controls, with no significant differences in age (P = 0.121), gender (P = 0.537) found between three groups. As well, no differences regards DM (P = 0.06), smoking (P = 0.092), TSH (P = 0.332), free T3 (P = 0.789), free T4 (P = 0.434), FBS (P = 0.353), 2hs PPBS (P = 0.198), TC (P = 0.413), TG (P = 0.777), LDL (P = 0.157), HDL (P = 0.214), Creatinine (P = 0.087), Urea (P = 0.194), Hb (P = 0.593), CRP (P = 0.150) were detected between the three groups. We found significantly higher mean systolic blood pressure (SBP) in the hypertensive group (mean ±SD = 166.2 ± 21.32 mmHg) than non-hypertensive group (mean ± SD = 139.2 ± 19.39 mmHg) and controls (mean ±SD = 137.33 ± 12.95 mmHg), but insignificant mean DBP (P = 0.099). The full information of these characteristics was exhibited in Table 2. For patients with CVS, higher NIHSS (at admission and after 72 h, P < 0.001 for each) and mRS (on discharge*,* P = 0.037) scores in the non-hypertensive group.

**Table 2:** Bivariant analysis of basic characteristics and laboratory parameters of the three studied groups.

Parameter		Acute CVS patients with HTN (n = 91)		Acute CVS non-hypertensive patients (n = 90)		Control (n = 51)		P-value
		n	%	n	%	n	%	
**Sex**	**Female**	19	20.87%	18	20.0%	11	21.57%	0.537^a^
	**Male**	72	79.13%	72	80.0%	40	78.43%	
**Diabetic**	**Yes**	27	29.68%	10	11.11%	13	25.5%	0.06^a^
	**No**	64	70.32%	80	88.89%	38	74.5%	
**Smoking**	**Yes**	31	34.06%	49	59.3%	21	41.17%	0.092^a^
	**No**	60	65.94%	41	40.7%	30	58.83%	

**Parameter**		**mean**	**± SD**	**mean**	**± SD**	**mean**	**± SD**	**P-value**
**Age (years)**		59.4	8.55	56.71	11.53	55.66	10.08	0.121^b^
**Thyroid functions**	**TSH (uIU/ml)**	2.34	1.35	3.04	1.03	2.11	0.97	0.332^b^
	**free T3 (pg/ml)**	2.33	1.09	2.51	1.11	2.49	1.33	0.789^b^
	**free T4 (ng/dl)**	1.85	0.43	1.73	0.3	1.93	0.29	0.433^b^
**Blood Glucose(mg/dl)**	**FBG (mg/dl)**	110.35	39.8	109.55	18.9	111.24	15.33	0.353^b^
	**2hs PPBG (mg/dl)**	184.65	59.25	149.36	42.25	150.15	13.33	0.198^b^
**Lipid profile (mg/dl)**	**TC (mg/dl)**	194.88	39.91	171.33	40.25	180.69	33.58	0.413^b^
	**TG (mg/dl)**	150.35	35.8	160.08	41.25	153.66	37.12	0.777^b^
	**LDL (mg/dl)**	121.25	29.33	113.15	33.33	119.05	37.25	0.157^b^
	**HDL (mg/dl)**	33.9	11.21	30.9	8.61	40.71	10.22	0.214
**Blood pressure (mmHg)**	**SBP mmHg**	166.2	21.32	139.2	19.39	137.33	12.95	**0.04∗**^b^
	**DBP mmHg**	94.55	11.88	82.37	8.61	85.75	7.45	0.099^b^
**Renal functions(mg/dl)**	**S. creatinine (mg/dl)**	1.29	0.47	1.03	0.36	1.13	0.29	0.087^b^
	**B. urea (mg/dl)**	44.23	17.58	47.33	13.63	41.97	10.28	0.194^b^
**Hb (g/dl)**		14.51	2.33	13.93	3.82	14.09	3.66	0.593^b^
**NIHSS**	**At admission**	10.85	3.09	14.57	5.64	0.00	0.00	**<0.001∗**^b^
	**After 72 hs**	3.55	1.99	8.64	3.01	0.00	0.00	**<0.001∗**^b^
**mRS**	**on Discharge**	2.50	1.70	3.8	0.63	0.00	0.00	**0.037∗**^b^

CVS, cerebrovascular stroke; TSH, thyroid stimulating hormone; T3, triiodothyronine; T4, thyroxine, FBG, fasting blood glucose; 2hsPPBG, 2 h postprandial blood glucose; TC, total cholesterol; TG, triglycerides; LDL, low-density lipoprotein, HDL, high-density lipoprotein; SBP, systolic blood pressure, DBP, diastolic blood pressure, Hb, hemoglobin; CRP, C-reactive protein; NIHSS, National Institutes of Health Stroke Scale; mRS, modified Rankin Scale.

a Chi-square (χ2),

b one-way ANOVA.

### 3.2 Statistical analysis of the presence of other risk factors and comorbidities in acute CVS patients with HTN and acute CVS non-hypertensive patients

By comparing the two patient groups, it was found that the hypertensive group had a significantly higher incidence of IHD than the non-hypertensive group (P = 0.003). There were no significant differences between the two groups regards diabetes, smoking, H/O stroke, atherosclerosis, stenosis, AF, RHD, or valve replacement (Table 3).

**Table 3:** Statistical analysis of the presence of other risk factors and comorbidities in Acute CVS patients with HTN and Acute CVS non-hypertensive patients.

Parameter		CVS-HTN (n = 91)		CVS-NHTN (n = 90)		P-value Chi-square (χ2)
		n	%	n	%	
**Diabetic**	**Yes**	27	29.68%	10	11.11%	0.053
	**No**	64	70.32%	80	88.89%	
**Smoking**	**Yes**	31	34.06%	49	59.3%	0.269
	**No**	60	65.94%	41	40.7%	
**H/O Stroke**	**Yes**	12	13.18%	12	13.33%	0.998
	**No**	79	86.82%	78	86.67%	
**Atherosclerosis**	**Yes**	39	42.86%	32	35.55%	0.133
	**No**	52	57.14%	58	54.45%	
**Carotid stenosis**	**Yes**	10	10.98%	15	16.67%	0.281
	**No**	81	89.02%	75	83.33%	
**AF**	**Yes**	13	14.28%	9	10.00%	0.301
	**No**	78	85.72%	81	90.00%	
**IHD**	**Yes**	41	45.06%	10	11.12%	**0.003∗**
	**No**	50	54.94%	80	88.88%	
**RHD**	**Yes**	3	3.30%	11	12.22%	0.071
	**No**	88	96.70	79	87.78%	
**Valve Replacement**	**Yes**	1	1.10%	2	2.22%	0.853
	**No**	90	98.90%	88	97.78	

CVS-HTN, cerebrovascular stroke in hypertensive patients; CVS-NHTN, cerebrovascular stroke in non-hypertensive patients; H/O stroke, history of stroke, AF, atrial fibrillation, IHD, ischemic heart diseases; RHD, rheumatic heart diseases.

### 3.3 Comparison of *NEAT1*, *HOTAIR*, and *GAS5* levels in the sera of the three studied groups (CVS + HTN, CVS + NHTN, and controls)

**Fig. 2:**
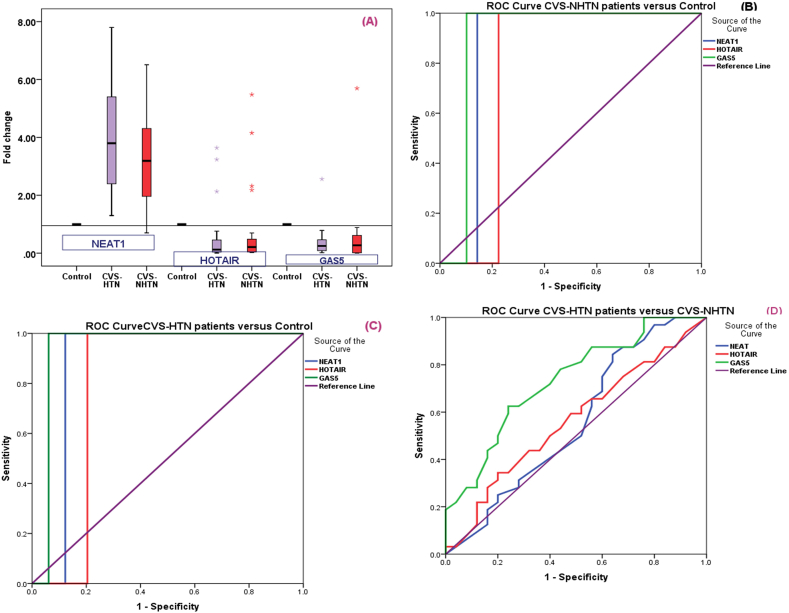
**A;** The serum lncRNAs; *NEAT1*, *HOTAIR*, and *GAS5* levels among the three studied groups (CVS + HTN, CVS + NHTN, and controls). **B;** ROC curve analysis for *NEAT1, HOTAIR,* and *GAS5* in CVS non-hypertensive patients versus controls. **C;** ROC curve analysis for *NEAT1, HOTAIR,* and *GAS5* in CVS-hypertensive patients versus controls. **D;** ROC curve analysis for *NEAT1, HOTAIR,* and *GAS5* in CVS-hypertensive patients versus CVS non-hypertensive patients.

When we examined the serum lncRNAs; *NEAT1*, *HOTAIR*, and *GAS5* levels among the three studied groups (CVS + HTN, CVS + NHTN, and controls) we found that there was a higher level of *NEAT1* in each case group (median (IQR) = 3.68 (1.35–7.35) for hypertensive group and median (IQR) = 3.05 (0.95–6.45) for the non-hypertensive group when compared to controls, and stroke patients with hypertension had significant higher *NEAT1* when compared to stroke patients without hypertension (P = 0.04). Regarding *HOTAIR,* it was significantly downregulated in all case groups (medians in CVS + HTN and CVS + NHTN patients were 0.13, 0.34 respectively) when compared to controls with a significantly lower level in the hypertensive group (P = 0.05). Serum *GAS5* was significantly lower in patients’ groups (median (IQR) = 0.16 (0.02–0.55) for hypertensive group and median (IQR) = 0.25 (0.03–0.99) for non-hypertensive group compared to controls with significant lower level in hypertensive group (*p* = 0.02) (Table 4, Fig. 2A).

**Table 4:** Statistical analysis (Mann–Whitney-U test) of the *NEAT1, HOTAIR* and *GAS5* fold change levels among the three studied groups.

Marker	CVS**-**HTN			CVS**-N**HTN			P-value
	Median	IQR		Median	IQR		
*NEAT1*	3.68	1.35	7.35	3.05	0.95	6.45	0.04^a^∗
							<0.0001^b^∗
							<0.000 c∗
*HOTAIR*	0.13	0.04	0.81	0.34	0.04	0.71	0.05^a^∗
							<0.001^b^∗
							<0.000 c∗
*GAS5*	0.16	0.02	0.55	0.25	0.03	0.99	0.02^a^∗
							<0.0001^b^∗
							<0.000 c∗

Fold change levels of serum target non-coding RNA expressions relative to controls that were calculated using 2^−ΔΔCT^. Control fold change levels are equivalent to 1.

a Comparison of cerebrovascular stroke in hypertensive patients versus cerebrovascular stroke in non-hypertensive patients.

b Comparison of cerebrovascular stroke in non-hypertensive patients versus controls.

c Comparison of cerebrovascular stroke in non-hypertensive patients versus controls.

### 3.4 Statistical analysis of the levels of the *NEAT1*, *HOTAIR*, and *GAS5* regarding patients’ characteristics and some clinical data among patients of acute CVS-HTN and acute non-CVS-HTN groups

Results reported in (Table 5) showed that significant higher *NEAT1* was associated with the presence of diabetes mellitus and history of stroke (in both patient groups), but lower *HOTAIR* was significantly associated with patients with a history of stroke in the hypertensive group (P = 0.05) as well as lower *GAS5* was significantly associated with diabetic patients in hypertensive group (P = 0.04).

**Table 5:** Statistical analysis of the levels of the NEAT1, HOTAIR, and GAS5 regarding patients’ characteristics and some clinical data among patients of Acute CVS-HTN and acute non-CVS-HTN groups (Mann–Whitney-U test).

Parameters		CVS-HTN (n = 91)						CVS-NHTN (n = 90)					
		NEAT1		HOTAIR		GAS5		NEAT1		HOTAIR		GAS5	
		Median (IQR)	P	Median (IQR)	P	Median (IQR)	P	Median (IQR)	P	Median (IQR)	P	Median (IQR)	P
**Sex**	**F**	3.78 (1.29–6.48)	0.76	0.13(0.05–1.06)	0.09	0.17(0.01–0.72)	0.16	3.15(0.83–6.45)	0.58	0.35(0.04–1.08)	0.11	0.26(0.03–0.79)	0.13
	**M**	3.59 (1.35–7.35		0.14(0.04–0.81)		0.16(0.03–0.61)		3.05(0.96–7.05)		0.34(0.03–0.83)		0.24(0.04–1.01)	
**DM**	**yes**	4.37 (2.58–10.45)	**0.03∗**	0.14(0.03–0.67)	0.06	0.11(0.02–0.77)	**0.04**∗	5.01(2.01–10.45)	**0.02∗**	0.31(0.05–1.03)	0.052	0.24(0.03–1.03)	0.06
	**no**	2.01 (0.78–5.35)		0.12(0.04–1.03)		0.16(0.05–0.79)		3.25(0.98–6.55)		0.35(0.03–0.98)		0.26(0.02–0.98)	
**Smoking**	**yes**	3.68(1.45–7.21)	0.51	0.13(0.05–0.79)	0.11	0.16(0.03–0.82)	0.19	3.14(1.03–8.96)	0.63	0.34(0.03–1.02)	0.07	0.26(0.03–1.01)	0.09
	**no**	3.59(1.05–8.06)		0.12(0.04–1.01)		0.17(0.01–1.06)		3.09(0.98–7.21)		0.33(0.04–0.89)		0.25(0.04–0.98)	
**H/O Stroke**	**yes**	5.33(3.04–8.95)	**0.03∗**	0.11(0.02–0.69)	**0.05∗**	0.15(0.03–0.79)	0.06	4.99(2.42–8.57)	**0.04∗**	0.33(0.02–1.04)	0.09	0.24(0.02–1.03)	0.07
	**no**	2.35(0.86–5.64)		0.18(0.04–1.05)		0.16(0.02–0.91)		2.09(0.97–5.08)		0.34(0.03–0.79)		0.26(0.03–0.98)	
**Atheros**	yes	3.75(2.54–9.07)	0.70	0.14(0.06–0.68)	0.17	0.16(0.03–0.88)	0.65	3.09(0.98–6.78)	0.62	0.34(0.05–0.91)	0.14	0.22(0.03–1.41)	0.13
	no	3.49(1.55–7.24)		0.13(0.04–0.95)		0.16(0.02–0.57)		3.15(1.09–8.77)		0.33(0.04–1.03)		0.25(0.04–0.99)	
**Stenosis**	yes	4.02(1.55–7.33)	0.55	0.14(0.04–0.83)	0.06	0.15(0.04–0.71)	0.17	3.01(1.08–7.44)	0.43	0.37(0.03–0.82)	0.15	0.25(0.03–1.07)	0.09
	no	3.44(1.88–10.14)		0.12(0.03–0.92)		0.17(0.02–0.64)		3.25(1.23–8.62		0.33(0.06–0.91)		0.24(0.04–0.097)	
**AF**	yes	3.77(1.35–8.02)	0.40	0.13(0.025–0.80)	0.06	0.16(0.03–0.95)	0.56	3.22(1.09–8.33)	0.79	0.35(0.06–0.92)	0.11	0.27(0.01–1.07)	0.06
	no	3.29(1.02–9.01)		0.12(0.04–0.69)		0.16(0.04–0.62)		3.05(0.98–7.66)		0.34(0.03–0.88)		0.25(0.04–0.89)	
**IHD**	yes	3.59(1.32–7.99)	0.56	0.14(0.02–1.01)	0.09	0.17(0.03–0.77)	0.09	3.20(0.95–6.08)	0.38	0.36(0.03–0.86)	0.07	0.24(0.01–0.99)	0.23
	no	3.45(1.41–8.67)		0.12(0.04–0.89)		0.16(0.04–0.93)		3.05(1.09–7.58)		0.33(0.04–0.79)		0.26(0.04–0.93)	
**RHD**	yes	3.71(1.29–8.74)	0.63	0.13(0.03–0.91)	0.07	0.16(0.02–1.05)	0.39	3.88(0.95–7.54)	0.75	0.35(0.03–0.97)	0.09	0.23(0.04–1.06)	0.16
	No	3.45(1.33–9.25)		0.12(0.05–1.06)		0.17(0.03–0.81)		3.01(1.01–8.99)		0.34(0.03–0.73)		0.25(0.03–0.76)	
**VR**	**Yes**	3.25(2.55–8.10)	0.49	0.13(0.03–0.95)	0.13	0.15(0.01–0.71)	0.13	3.01(1.08–8.66)	0.68	0.35(0.03–0.89)	0.31	0.26(0.03–1.03)	0.07
	**No**	3.69(1.33–7.55)		0.12(0.04–0.88)		0.14(0.02–0.61)		3.21(0.97–7.22)		0.34(0.03–0.81)		0.25(0.04–0.97)	

DM, diabetes mellitus; H/O stroke, history of stroke; Atheros, atherosclerosis; AF, atrial fibrillation, IHD, ischemic heart diseases, RHD, rheumatic heart diseases; VR, valve replacement.

### 3.5 Spearman correlations of *NEAT1, HOTAIR*, and *GAS5* levels and different parameters in hypertensive and non-hypertensive CVS patients

**Fig. 3:**
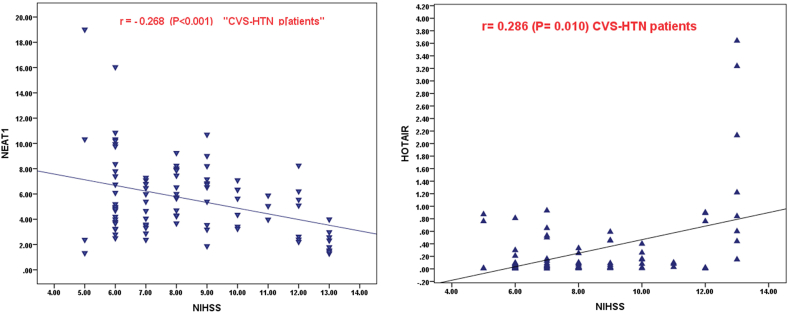
SPSS Scatter dot graph represents the Spearman correlation of NEAT1 and HOTAIR, with NIHSS in the CVS-HTN patients' group.

**Fig. 4:**
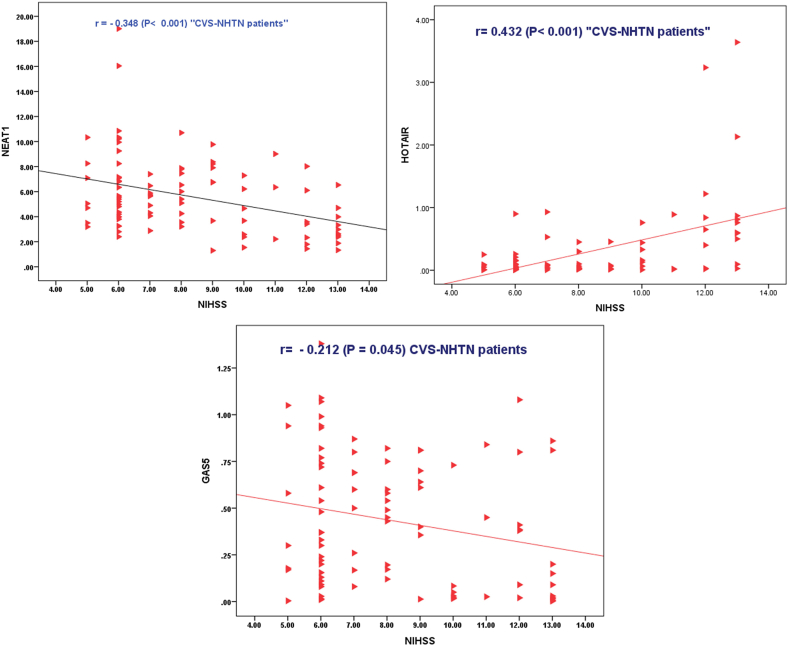
SPSS Scatter dot graph represents the Spearman correlation of NEAT1, HOTAIR, and GAS5 with NIHSS in the CVS-NHTN patients' group.

The results of the correlational analysis are shown in (Table 6, Fig. 3, Fig. 4), The most important results are the significant positive correlation between *NEAT1* and *GAS5* (*r* = 0.458, P = 0.001 in hypertensive group and r = 0.687, P < 0.001 in non-hypertensive group). and the significant negative correlation of each with *HOTAIR* in both patients’ groups (for *NEAT1*; *r* = -0.790, P < 0.001 in HTN, and *r=*- 0.774, P < 0.001 in NHTN, for *GAS5; r* = -0.526*,* P < 0.001 in HTN*, and r=*-0.554, P < 0.001 in NHTN*)* Also, the detected significant negative correlation of each *NEAT1* (*r* = -0.268, P = 0.010 in HTN and *r* = -0.348, P = 0.001 in NHTN group) or *GAS5 (r=*- 0.212, P < 0.045 in NHTN) with NIHSS score while the significant positive correlation between *HOTAIR* and NIHSS score r = 0.286, P = 0.010 in HTN, and r = 0.432, P < 0.001 in NHTN). Besides the reported significant negative correlation between NEAT1 with SBP while a significant negative correlation between *HOTAIR* and SBP or DBP. Moreover, there was a significant positive correlation between NEAT1 and HDL in both groups (*r* = 0.219, P = 0.037 in HTN and *r* = 0.302, P = 0.002 in NHTN group) and between GAS5 and HDL in NHTN (*r = *0.225, P = 0.015), but, a significant negative correlation between HOTAIR and HDL in NHTN (*r = *−0.233, P = 0.023). Taken together, these results suggest that *NEAT1* and *GAS5* are defensive lncRNAs while *lncHOTAIR* is a risky lncRNA.

**Table 6:** Spearman Correlation of NEAT1, HOTAIR, and GAS5 levels with different parameters in hypertensive CVS patients and non-hypertensive CVS patients.

Variable		CVS-HTN (n = 91)			CVS-NHTN (n = 90)		
		NEAT1	HOTAIR	GAS5	NEAT1	HOTAIR	GAS5
**NEAT1**	r		**- 0.790∗**	**0.458∗**		**- 0.774∗**	**0.687∗**
	P		**<0.001**	**0.001**		**<0.001**	**<0.001**
**HOTAIR**	r			**−0.526**∗			**−0.554∗**
	P			**<0.001**			**<0.001**
**Age (years)**	r	0.089	- 0.109	−0.071	0.039	- 0.019	- 0.094
	P	0.533	0.421	0.593	0.657	0.754	0.635
**SBP (mmHg)**	r	−0.072	**0.280∗**	−0.199	**−0.243∗**	**0.324∗**	−0.121
	P	0.498	**0.022**	0.061	**0.021**	**0.002**	0.256
**DBP (mmHg)**	r	−0.128	**0.219∗**	−0.177	- 0.170	**0.270∗**	- 0.162
	P	0.103	**0.037**	0.08	0.07	**0.010**	0.128
**NIHSS Score at 24 hs**	r	**- 0.268∗**	**0.286∗**	−0.123	**- 0.348∗**	**0.432∗**	**- 0.212∗**
	P	**0.010**	**0.010**	0.247	**0.001**	**<0.001**	**<0.045**
**Hb (g/dl)**	r	−0.111	0.172	0.113	- 0.108	−0.145	−0.113
	P	0.487	0.092	0.570	0.629	0.211	0.570
**B. urea (mg/dl)**	r	−0.077	0.109	0.088	0.119	0.095	0.201
	P	0.663	0.091	0.527	0.543	0.614	0.084
**S. creatinine (mg/dl)**	r	−0.081	−0.196	0.097	0.081	−0.071	−0.117
	P	0.161	0.155	0.487	0.657	0.633	0.109
**FBG (mg/dl)**	r	0.044	- 0.106	- 0.019	−0.019	0.118	−0.176
	P	0.601	0.214	0.778	0.840	0.553	0.207
**2hs PPBG (mg/dl)**	r	0.060	−0.203	0.097	0.075	−0.094	−0.103
	P	0.667	0.069	0.681	0.609	0.687	0.209
**TC (mg/dl)**	r	0.176	0.182	−0.136	0.119	−0.155	- 0.116
	P	0.204	0.088	0.537	0.453	0.107	0.104
**TG (mg/dl)**	r	0.077	- 0.119	0.171	0.129	0.111	−0.201
	P	0.633	0.221	0.108	0.591	0.601	0.0.74
**LDL (mg/dl)**	r	−0.139	- 0.059	−0.044	−0.118	0.159	- 0.107
	P	0.157	0.701	0.735	0.257	0.103	0.234
**HDL (mg/dl)**	r	**0.219∗**	−0.201	**0.225∗**	**0.302∗**	**−0.233∗**	0.199
	P	**0.037**	0.057	**0.015**	**0.002**	**0.023**	0.061
**TSH (IU/ml)**	r	−0.091	0.133	−0.176	−0.121	0.182	0.123
	P	0.440	0.253	0.207	0.387	0.071	0.388
**Free T3 (pg/ml)**	r	0.175	−0.094	0.109	0.177	0.129	0.188
	P	0.087	0.687	0.211	0.161	0.118	0.109
**Free T4 (ng/dl)**	r	−0.091	0.137	−0.155	0.019	−0.095	0.108
	P	0.544	0.213	0.101	0.840	0.733	0.833

CVS-HTN, cerebrovascular stroke in hypertensive patients; CVS-NHTN, cerebrovascular stroke in non-hypertensive patients; TSH, thyroid stimulating hormone; T3, triiodothyronine; T4, thyroxine, FBG, fasting blood glucose; 2hsPPBG, 2 h postprandial blood glucose; TC, total cholesterol; TG, triglycerides; LDL, low-density lipoprotein, HDL, high-density lipoprotein; SBP, systolic blood pressure, DBP, diastolic blood pressure, Hb, hemoglobin; CRP, C-reactive protein; NIHSS, National Institutes of Health Stroke Scale; mRS, modified Rankin Scale.

### 3.6 Multivariant linear regression analysis regarding CVS-HTN patients and CVS-NHTN patients with NIHSS score as a dependent factor

**Fig. 5:**
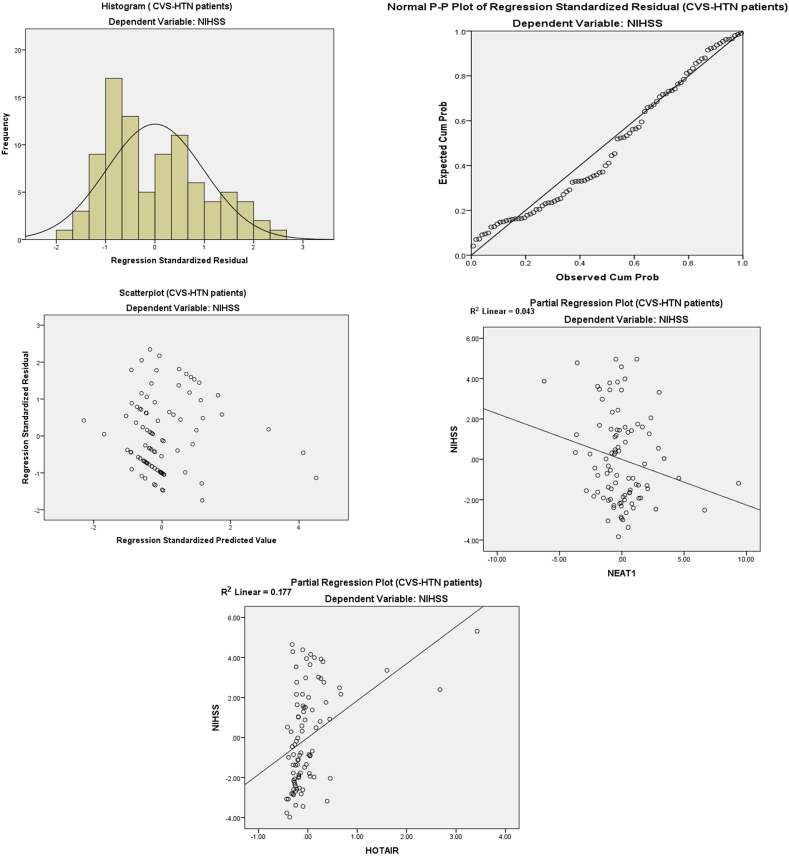
Linear regression histogram, P P plot, and partial regression plot for the fold changes in NEAT1, HOTAIR, and GAS5 in hypertensive patients with cerebrovascular stroke.

**Fig. 6:**
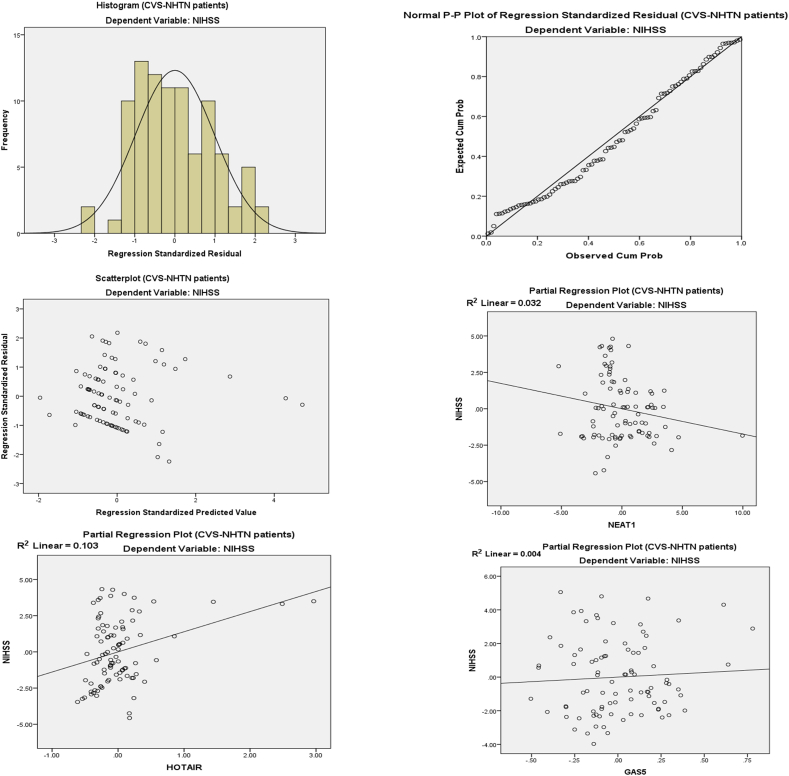
Linear regression histogram, P P plot, and partial regression plot for the fold changes in NEAT1, HOTAIR, and GAS5 in a non-hypertensive patient with cerebrovascular stroke.

On Multi-variant Analysis (Table 7 A & B and Fig. 5, Fig. 6) with dependent variables NEAT1, HOTAIR, and GAS5 in the corrected model, the adjusted R squared for the hypertensive group is 0.263 with significant F change (11.592) with P < 0.001 and adjusted R squared for the non-hypertensive group is 0.195 with significant F change (8.281) with P < 0.001.

**Table 7:** (A&B): Multivariant linear regression analysis regarding CVS-HTN patients and CVS-NHTN patients with NIHSS score as a dependent factor.

Table 7A						
Group	R	R Square	Std. Error of the Estimate	Change Statistics		
				R Square Change	F Change	Sig. F Change
**CVS-HTN**	0.537^a^	0.288	2.23466	.288	11.592	<0.001
**CVS-NHTN**	0.471^a^	0.222	2.14325	.222	8.281	<0.001

Table 7**B**							
**CVS-HTN (n = 91)**	Unstandardized Coefficients		Standardized Coefficients	t	P	Collinearity Statistics	
	B	S.E.	Beta				
						Tolerance	VIF
**NEAT1**	−0.226	0.116	−0.265	−1.956	**0.050**	.452	2.211
**HOTAIR**	1.846	0.430	0.419	4.297	**<0.001**	.870	1.149
**GAS5**	0.544	0.976	0.073	.557	0.579	.487	2.054
**Constant**	8.764	0.578		15.168	**< 0.001**		

**CVS-NHTN (n=90)**	Unstandardized Coefficients		Standardized Coefficients	t	P	Collinearity Statistics	
	B	S.E.	Beta				
						Tolerance	VIF
**NEAT1**	−0.174	0.102	−0.220	−1.708	0.091	.538	1.860
**HOTAIR**	1.191	0.440	0.342	2.159	**0.02**	0.661	1.014
**GAS5**	0.335	0.798	0.048	0.420	0.675	0.674	1.483
**Constant**	8.809	0.603		14.618	**< 0.001**		

a Predictors, (Constant) GAS5, HOTAIR, NEAT1. Dependent Variable: NIHSS.

By testing the fold change in NEAT1, HOTAIR, and GAS5 (Constant) probability to be predictors for cerebrovascular stroke, in hypertensive cerebrovascular stroke patients the Standardized Coefficients (Beta) are −0.265, 0.419, and 0.073 respectively and the P value is 0.050, <0.001 and 0.579 respectively. On testing the fold change in NEAT1, HOTAIR, and GAS5 (Constant) probability to be predictors for cerebrovascular stroke, in non-hypertensive cerebrovascular stroke patients, the Standardized Coefficients (Beta) are −0.220, 0342, and 0.048 respectively with a P value of 0.091, 0.02 and 0.675 respectively.

### 3.7 ROC curve analysis for *NEAT1, HOTAIR,* and *GAS5* in the three studied groups

ROC curve analysis for the *NEAT1*, *HOTAIR*, and *GAS5* showed statistically significant differences in CVS hypertensive patients and CVS non-hypertensive patients compared to control, with cut-off points of 1.39, 0.68, and 0.82, respectively, with a sensitivity of 94.6, 81.5, and 96.8%, respectively, and specificity of 100% for the three markers in CVS hypertensive patients, and cut-off points 1.33, 0.75 0.84, respectively, sensitivity 91.2, 75.8, and 91.9%, respectively, specificity of 100% for the three markers in CVS non-hypertensive patients, as shown in (Table 8, Fig. 2B and C).

**Table 8:** ROC curve analysis for NEAT1, HOTAIR, and GAS5 in the CVS-HTN, CVS-NHTN patients, and the healthy control groups.

CVS-HTN patients vs. control						
LncRNA	AUC 95% CI	P-value	Cut-off point	Sensitivity (%)	Specificity (%)	Total accuracy
***NEAT1***	0.878 (0.786–0.969)	**<0.0001**	1.39	94.6	100	97.3%
***HOTAIR***	0.796 (0.683–0.909)	**<0.0001**	0.68	81.5	100	90.75
***GAS5***	0.939 (0.872–1.000)	**<0.0001**	0.82	96.8	100	98.4

**CVS-NHTN patients vs. control**						
**LncRNA**	**AUC** **95% CI**	**P-value**	**Cut-off point**	**Sensitivity (%)**	**Specificity (%)**	**Total accuracy**
***NEAT1***	0.857 (0.759–0.955)	**<0.0001**	1.33	91.2	100	95.6
***HOTAIR***	0.776 (0.659–0.892)	**<0.0001**	0.75	75.8	100	87.9
***GAS5***	0.898 (0.813–0.983)	**<0.0001**	0.84	91.9	100	95.95

**CVS-HTN patients vs. CVS-NHTN patients**						
**LncRNA**	**AUC** **95% CI**	**p-value**	**Cut-off point**	**Sensitivity (%)**	**Specificity (%)**	**Total accuracy**
***NEAT1***	0.585 (0.402–0.715)	0.455	3.83	86.4	55.95	71.17
***HOTAIR***	0.562 0.411–0.713)	0.426	0.47	65.3	59.14	62.22
***GAS5***	0.733 (0.603–0.863)	**0.003**	0.37	81.68	69.77	75.72

ROC curve analysis for *GAS5* in CVS hypertensive patients compared with CVS non-hypertensive patients revealed a statistically significant difference (P = 0.003) with a cut-off point of 0.37 with 81.68% sensitivity and 69.77% specificity. while, ROC curve analysis for *NEAT1*, *HOTAIR* in CVS hypertensive patients compared with CVS non-hypertensive patients revealed a statistically nonsignificant difference with a cut-off point of 3.83 and 0.47, with a sensitivity of 86.4 and 65.3%, respectively, and specificity of 55.95 and 59.14%, respectively (Table 8, Fig. 2D).

## 4 Discussion

CVS is a serious disease with fatal consequences that necessitates immediate medical attention for the best possible outcomes [1]. The most common risk factor for stroke is hypertension, which has been observed in approximately 64% of stroke patients [3]. Recently, there has been renewed interest in lncRNAs that may aid in the early detection of stroke risk and serve as a therapy target [5]. LncRNAs NEAT1, GAS5, and HOTAIR have previously been linked to the risk and development of CVS, and to pathologic vasculature remodeling in response to hypertension, which is the primary risk factor for cerebrovascular dysfunction [5,12].

In reviewing the literature, no study was found to have explored these lncRNAs in patients with CVS with hypertension versus patients with CVS without hypertension. Therefore, the present study set out with the aim to compare expressions of three lncRNAs (*NEAT1, GAS5,* and *HOTAIR*) between three groups (hypertensive patients with acute CVS, non-hypertensive patients with CVS, and a control group), as well as their association with clinicopathological data and disease severity score.

Concerning the first research question, it was discovered that patients in each case group had statistically higher levels of NEAT1 and lower levels of HOTAIR and GAS5 compared to control levels, with higher significant NEAT1 but lower significant HOTAIR and GAS5 in the hypertensive group. Furthermore, we found that NEAT1 and GAS5 expression was significantly negatively correlated with NIHSS score, suggesting a possible protective role. While HOTAIR was significantly positively correlated with NIHSS score, indicating its harmful effects. Another significant finding was that NEAT1 was significantly negatively correlated with DBP, whereas HOTAIR was significantly positively correlated with SBP and DBP.

The possible explanations for these findings are as follows: (a) In two 2022 studies that documented the beneficial role of NEAT1 in the induced mice injury model, they revealed that induced NEAT1 expression inhibits inflammasome activation by NLRP3 in microglia, alleviating the negative outcomes of ischemic stroke [26]. Also, by activating Sirt3, upregulated NEAT1 reduces oxidative stress and apoptosis caused by oxygen-glucose deprivation/reperfusion (OGD/R) [27]. Similarly, Zhou et al., 2019 revealed that NEAT1 overexpression in oxygen-glucose deprivation (OGD)-induced brain microvascular endothelial cells (BMECs) promotes hypoxic brain cell viability and enhances angiogenesis to restore blood flow by inhibiting miR-377 and upregulating the expression of VEGFA, SIRT1, and BCL-XL [28]. As a result, it helped to restore normal cerebrovascular physiology, which resulted in less disease severity and, as a result, a lower NIHSS score. Endothelial cells (EC) dysfunction rat model increased NEAT1 expression, and overexpression of NEAT1 increased viability but decreased apoptotic rates of EC by inhibiting oxidative stress-induced vascular EC injury by activating the miR-181d-5p/CDKN3 axis [29]. Thus, increasing NEAT1 in the hypertensive group may imply a protective role against hypertension-induced stroke.

(b) Our findings broadly support the findings of another study in this area linking GAS5 to myocardial infarction (MI), which found reduced GAS5 transcript levels in the hearts of MI-modeled mice [30], and that induced GAS5 may be able to reduce cardiomyocyte apoptosis caused by MI by downregulating Semaphorin (sema3a), a secretory protein that could reduce inflammation and improve cardiac function after MI by promoting inflammation resolution [9]. Correia et al., 2021 also reported that treadmill aerobic exercise improved contractility and cardiac function in rats after MI by normalizing H19, MIAT, and GAS5 expression levels [31]. Thus, GAS5 is a protective lncRNA that aids in the resolution of inflammation and, as a result, improves cognitive and neuronal functions, leading to higher NIHSS scores. Lower GAS5 levels in the hypertensive group could be explained by the fact that GAS5 was discovered to be primarily expressed in endothelial cells/vascular smooth muscle cells (ECs/VSMCs) and its expression was significantly downregulated in hypertension; additionally, GAS5 knockdown exacerbated hypertension-induced microvascular dysfunction by influencing several pathways such as EC multiplication, VSMC phenotypic transformation, and EC-VSMC interaction via β -catenin signaling [11]. Zhang et al., 2019 demonstrated that GAS5 inhibits PDGF-bb-induced VSMC proliferation and migration, in part by acting as a competitive endogenous RNA of miR21 and provide additional evidence that GAS5 may be a potential therapeutic candidate for hypertension [12]. The information in this paragraph strengthens the case for GAS5's role in both hypertension and ischemic stroke.

c) Regarding HOTAIR, elevated HOTAIR in permanent middle cerebral artery occlusion (pMCAO) mice brain tissues were significantly related to the larger infarcted area and worse neurological deficits and motor balance scores via the miR-148a-3p/KLF6 axis [32] or promotes ischemic infarct induced by hypoxia by up-regulating the expression of NADPH oxidase 2 (NOX2) enzyme which contributed to ROS formation [17]. As a result, HOTAIR aggravated the abnormal perceptive deficits and functional activities of CVS patients and increased the NIHSS score. HOTAIR was reduced in oxLDL-treated VSMCs, and its induction reduces their proliferative ability while increasing apoptosis via the miRNA-130b-3p/PPARα axis [33].

**Fig. 7:**
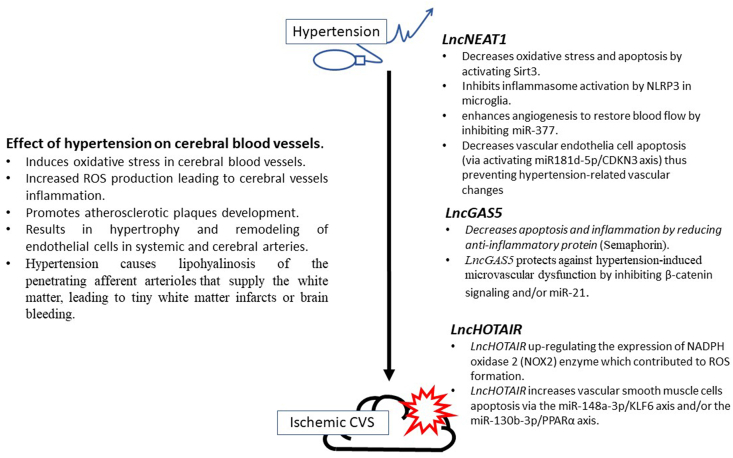
Proposed role of target lncRNAs in hypertension and, hypertension-induced ischemic stroke.

Based on the information presented above, we can conclude that by regulating the expression levels of these lncRNAs, we can target a critical pathway underlying both hypertension and hypertension-induced vascular occlusive diseases; a) Induction of NEAT1 can be beneficial by reducing oxidative stress (by activating Sirt3), relieving inflammation, (inhibiting inflammasome activation by NLRP3), enhancing angiogenesis and restoring blood flow (inhibiting miR-377 and upregulating the expression of VEGFA, SIRT1, and BCL-XL), and decreasing vascular endothelial cell apoptosis (via activating miR181d-5p/CDKN3 axis) thus preventing hypertension-related vascular changes b) GAS5 induction can aid in the resolution of vascular inflammation (by downregulating sema3a) and the prevention of hypertensive vascular remodeling (by inhibiting β-catenin signaling and/or miR-21). c) Conversely, silencing HOTAIR reduced ROS formation, vascular inflammation, and intimal apoptosis (via the miR-148a-3p/KLF6 axis and/or the miR-130b-3p/PPARα axis). These findings suggested that target lncRNAs could be used as a therapeutic target in hypertensive stroke patients (Fig. 7).

Limited clinical studies have demonstrated expressions of target lncRNAs in peripheral blood of CVS patients and linked their levels to clinical features of the disease as follows; a) For NEAT1, previous two studies have demonstrated increased NEAT1 in stroke patients compared to controls and not related to clinical categorical variables including hypertension but they found a positive correlation between *NEAT1* level and NIHSS score [6,34]. However, the current study's findings contradict previous research conducted by Zhou et al., 2022, who found that the NEAT1 level was decreased in CVS patients [26]. b) Only one study investigated GAS5 levels in CVS patients, and the study's findings contradict our findings, which found that GAS5 levels were increased in plasma samples collected from patients with acute stroke, and elevated GAS5 levels were positively correlated with NIHSS score and inflammatory cytokines [35]. c) Respects *HOTAIR*, no previous study explores its expression in stroke patients. While *Huang et al, 2021* reported a significant increase of *lnc-HOTAIR* in brain tissues of (pMCAO) mice with unfavorable outcomes and increasing neurological deficits and motor balance scores [36]. The correlation between three studied lncRNAs revealed a significant positive correlation between *NEAT1* and *GAS5* supporting the suggested protective roles, while a significant negative correlation between each of them and *HOTAIR* enforced their suggested opposite functions. In line with our findings, a previous study demonstrated a positive correlation between NEAT1 and GAS5 in breast cancer patients [23]. While Kamel et al., 2020 found no correlation between GAS5 and HOTAIR in patients with multiple sclerosis [22]. We also detected a significant positive correlation between NEAT1 or GAS5 and HDL, while, a significant negative correlation between HOTAIR and HDL. HDL level was found to be inversely correlated with stroke risk by removing cholesterol from blood stream thus decreasing the risk of atherosclerosis and its related occlusive vascular diseases [37].. Hence these findings enforce that NEAT1 and GAS5 are protective lncRNAs, but HOTAIR is a risky one.

ROC curve analysis for NEAT, HOTAIR, and GAS5 in CVS hypertensive patients versus CVS non-hypertensive patients revealed a statistically insignificant difference, with the exception of GAS5, which significantly can distinguish between stroke patients with hypertension and stroke patients without hypertension (cut-off point 0.37 with 81.68% sensitivity, 69.77% specificity). ROC curve analysis for the NEAT1, HOTAIR, and GAS5 revealed statistically significant differences in CVS patients, whether they had hypertension or not, when compared to controls, with fair sensitivity and high specificity, implying their diagnostic values.

For the first time, the findings presented here suggest that the *NEAT1, HOTAIR,* and *GAS5* are novel diagnostic and prognostic markers for stroke associated with hypertension. They could be used as novel therapeutic targets for hypertensive stroke patients by breaking the vicious cycle of oxidative stress, inflammation, and hypertension by inducing the production of NEAT1 and GAS5 while silencing HOTAIR could be a new therapeutic and preventive strategy aiding in the treatment of hypertension and preventing stroke formation in hypertensive patients. According to previous research, traditional antioxidative and anti-inflammatory drugs are of limited value in this case, implying that more specific interventions targeting the underlying mechanism of hypertension-induced stroke would be most beneficial [3].

**The study's limitations** include a relatively small sample of patients collected from the same area, so there is potential for patient selection bias. Due to the lack of a long-term follow-up program to evaluate the prognostic value of target lncRNAs and their relevance to recurrence probability, we recommend that future large-scaled multicentric studies be conducted to validate the current study's findings. Also, different polymorphisms in target lncRNAs and their relation to serum expression levels should be considered. In addition, to highlight the relevance of the current study's results to negative lncRNAs control whose circulating levels are not impacted by hypertension.

## 5 Conclusion

Patients in each case group had statistically higher levels of NEAT1 and lower levels of HOTAIR and GAS5 compared to control levels, with higher significant NEAT1 but lower significant HOTAIR and GAS5 in the hypertensive group. Furthermore, NEAT1 and GAS5 expression was significantly negatively correlated with NIHSS score, while HOTAIR was significantly positively correlated with NIHSS score. Another significant finding was that NEAT1 was significantly negatively correlated with DBP, whereas HOTAIR was significantly positively correlated with SBP and DBP. Therefore, lncRNAs NEAT1, HOTAIR, and GAS5 could be used as diagnostic and prognostic biomarkers of CVS that correlate with NIHSS score and could produce a novel target for CVS therapy.

## CRediT authorship contribution statement

**Marwa A. Ali:** Methodology, Investigation, Writing- original draft. **Olfat G. Shaker:** Conceptualization. **Abeer A. Khalifa:** Formal analysis. **Eman M. Ezzat:** Methodology, Investigation, Writing- original draft. **Hany Ahmed Elghobary:** Writing-original draft, Software, Methodology. **Tamer Sayed Abdel Mawla:** Writing, Review editing, Methodology, Investigation, and Supervision. **Ahmed Fathy Elkhateeb:** Writing, Review editing, Methodology, Investigation, and Supervision. **Ahmed Magdy Abdelrahman Elebiary:** Writing- review & editing, and Investigations. **Azza Mohamed Elamir:** Methodology, Investigation, Writing- original draft.

## Declaration of competing interest

The authors have no conflicts of interest.
